# Endometrial intraepithelial carcinoma in association with polyp: review of eight cases

**DOI:** 10.1186/1746-1596-8-25

**Published:** 2013-02-15

**Authors:** Masanori Yasuda, Tomomi Katoh, Shinichi Hori, Kimiyoshi Suzuki, Kisaku Ohno, Masanori Maruyama, Naruaki Matsui, Sayuri Miyazaki, Naoki Ogane, Yoichi Kameda

**Affiliations:** 1 Department of Pathology, Saitama Medical University International Medical Center, 1397-1 Yamane, Hidaka, Saitama 350-1298, Japan; 2 Seiwa Laboratory Company Limited, Saitama, Japan; 3 Health Sciences Research Institute East Japan Company Limited, Saitama, Japan; 4 Department of Obstetrics and Gynecology, Maruyama Memorial General Hospital, Saitama, Japan; 5 Department of Pathology, Tokai University Oiso Hospital, Kanagawa, Japan; 6 Division of Pathology, Ebina General Hospital, Kanagawa, Japan; 7 Division of Pathology, Kanagawa Cancer Center, Kanagawa, Japan

**Keywords:** Endometrial intraepithelial carcinoma (EIC), Endometrial polyp, Cytology

## Abstract

**Background:**

The uterine endometrial polyp (EMP) has a potential risk of developing malignant tumors especially in postmenopausal women. These malignancies include endometrial intraepithelial carcinoma (EIC).

**Patients and methods:**

Eight patients with EIC in the EMP, who were postmenopausal with ages ranging from 49 to 76 years (av. 62), were cytologically reviewed in comparison with histological findings.

**Results:**

The endometrial cytological findings were summarized as follows: mucous and watery diathesis as a background lacking or with little necrotic inflammatory changes; micropapillary cluster formation; abrupt transition between carcinoma cells and normal cells; nuclear enlargement; high N/C ratio; and single or a few prominent nucleoli. Histologically, one case had EIC alone in the EMP; three cases had EIC with stromal invasion confined to the EMP; and four cases had EIC in the atrophic endometrium in addition to EIC in the EMP. Seven patients have taken a disease-free course after surgical resection, but one patient died 44 months following the initial diagnosis because of the massive tumor extending over her peritoneal cavity.

**Conclusions:**

Endometrial cytology may be helpful for the detection of early endometrial adenocarcinomas with serous features including EIC. Some early stage endometrial adenocarcinomas represented by EIC exceptionally take an aggressive clinical course irrespective of a lack of extrauterine lesions.

**Virtual Slides:**

The virtual slide(s) for this article can be found here: http://www.diagnosticpathology.diagnomx.eu/vs/1651876760876449

## Introduction

The uterine body type II cancers are histogenetically distinguished from type I cancers with a background of glandular hyperplasia which is in association with the genetic alterations represented by PTEN inactivation [1]. Tumor development of uterine body serous adenocarcinoma, categorized as the type II group, has been clarified to be linked with a putative precursor lesion designated as endometrial intraepithelial carcinoma (EIC) [2-4]. EIC has been alternatively regarded as in situ serous adenocarcinoma [5,6] and is considered to usually occur in the setting of inactive or resting endometrium and frequently involves endometrial polyp (EMP) [3]. Many minimal serous adenocarcinomas, defined as limited to the endometrium and less than 1 cm [7], are also found to frequently have EIC and involve EMP [8]. However, the nomenclature of EIC remains controversial because there are morphological variations in the endometrial intraepithelial precancerous lesion, and EIC is known to be potentially complicated with the extrauterine lesion. Therefore, instead of EIC, a newly defined terminology of endometrial glandular dysplasia has been proposed [9]. Reportedly, 10–34% of endometrial cancers in postmenopausal women have been associated with EMPs [10]. Usually, the major adenocarcinomas are the endometrioid type and the second major adenocarcinomas are the serous type [10]. The eight cases with early endometrial carcinoma of the uterine body, characterized by EIC being associated with EMP, were subjected to close cytological review as well as histological review. For them, histological diagnostic confirmation was limited due to the inner location of tumors at the fundus and their small size. However, the endometrial cytological approach is supposed to be of help for the detection of these early lesions. The candidates of differential cytological diagnosis for these cases are advanced serous adenocarcinoma, clear cell adenocarcinoma, high grade endometrioid adenocarcinoma and also low grade endometrioid adenocarcinoma [11]. Immigration of ovarian carcinoma via the fallopian tube, instead of metastasizing to the endometrium, is also included in the differential diagnoses [12].

### Patients

The eight patients were postmenopausal, with ages ranging from 49 to 76 years (av. 62) when the cytological abnormalities were initially recognized. All of them visited their neighboring hospitals with a chief complaint of irregular genital bleeding. One patient had received surgical treatment for breast cancer 6 years before and had been administered an anti-estrogenic drug (Tamoxifen). There was no past history of other malignancies in the remaining patients. The endometrial cytology using Endocyte® (MSD Co., Tokyo, Japan) suspected adenocarcinoma which was consistent with being at the early stage, including EIC, for all the patients. However, the endometrial biopsy did not lead to the confirmation of malignancy except in two patients for whom the diagnosis was adenocarcinoma, although the adenocarcinoma could not be specified because the specimen was too small. They all underwent total abdominal hysterectomy and bilateral adnexectomy. Regional lymph node dissection was performed in three of the patients, resulting in no evidence of metastatic lesions. In two patients for whom polypectomy with hysteroscopic assistance had been performed previously, no residual carcinoma was found in the hysterectomy specimen.

## Results

**Figure 1:**
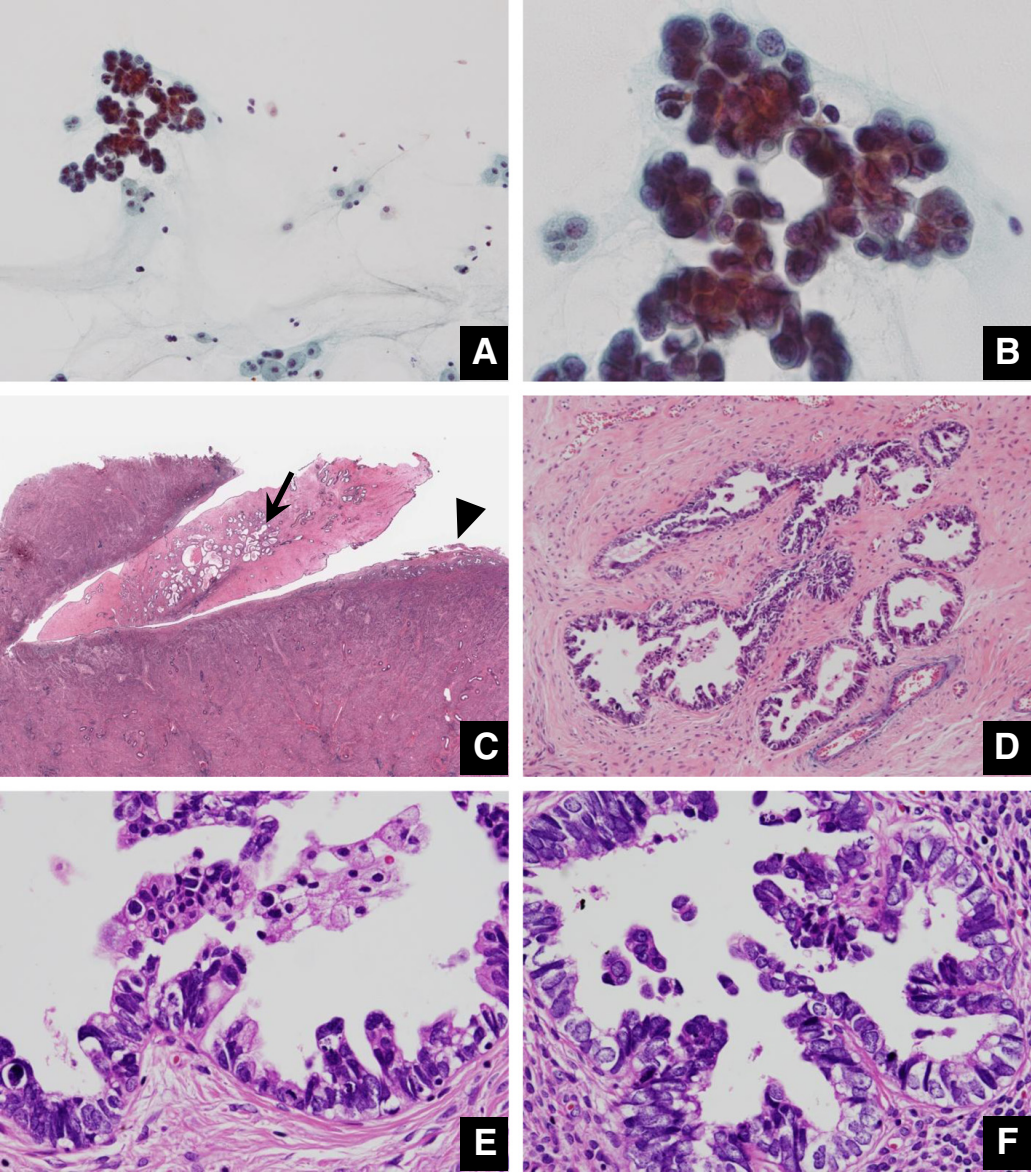
**Case 2 possessing EIC in both endometrial polyp and endometrium (A. Pap × 20, B. Pap × 60, C. loupe view, D. HE × 20, E. HE × 60, F. HE × 60). A.** Papillary-structured carcinoma cell cluster is accompanied by a watery background (Pap. ×20). **B.** The N/C ratio is fairly high and the chromatin is coarsely granular (Pap. ×60). **C.** Loupe findings show an endometrial polyp arising in the uterine fundus, measuring 1.5 cm in longitudinal length. The endometrium is markedly thin and atrophic. The area marked by an arrow and that marked by an arrowhead are depicted respectively in E and F. **D.** In the polyp, the glands composed of carcinoma cells are found to be aggregated in the fibrotic stroma. **E.** Carcinoma cells are replacing the glands in the polyp and stratified into micropapillary protrusions, lacking apparent stromal invasion (HE × 20). **F.** Carcinoma nests similar to those in the endometrial polyp (D) are also present in the endometrial gland (HE × 60).

**Figure 2:**
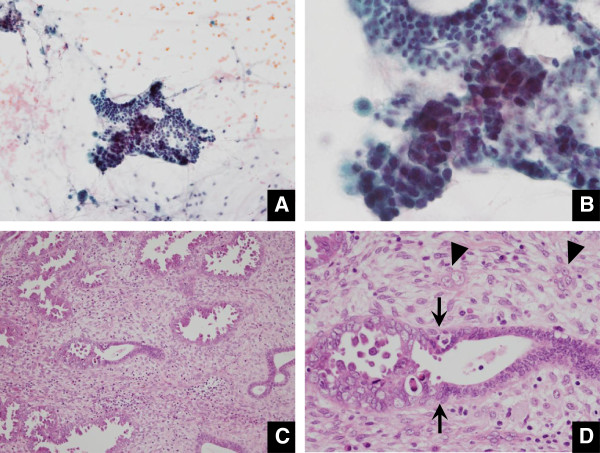
**Case 5 possessing EIC in both endometrial polyp and endometrium, but with stromal invasion in the polyp (A. Pap × 20, B. Pap × 60, C. HE × 20, D. HE × 60). A.** A carcinoma cell cluster is accompanied by a bloody background. **B.** Carcinoma cells having enlarged nuclei appear to be transitional with normal atrophic cells having small-sized nuclei (arrows). **C.** The polyp has many atypical glands with intraluminal papillary protrusion of the carcinoma cells. **D.** The border between the carcinoma cells and normal cells is clearly discernible (arrows). There are small carcinoma nests showing stromal invasion (arrowheads).

**Figure 3:**
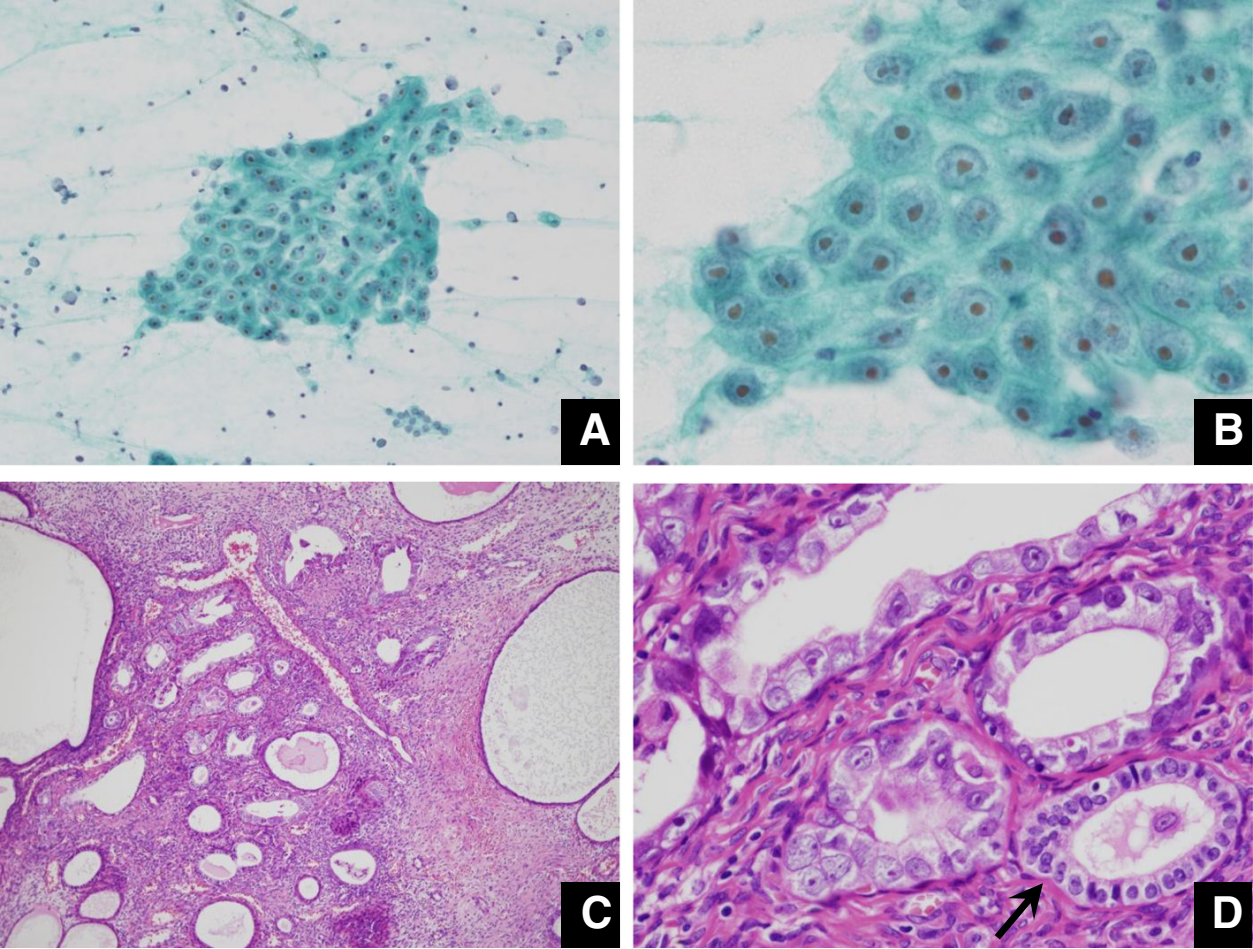
**Case 6 possessing EIC confined to endometrial polyp (A. Pap × 20, B. Pap × 60, C. HE × 60, D. HE × 60). A.** Large-sized carcinoma cell clusters are accompanied by a non-necroinflammatory background. **B.** Carcinoma cells mainly appear in a flat and sheet-like cluster. The nucleus has an enlarged eosinophilic nucleolus. **C.** The atypical glands are normal-sized, accompanied by cystic dilatation of atrophic normal glands. **D.** Carcinoma cells are replacing pre-existing glands and are arranged in a single layer (compare with normal gland marked by an arrow).

The EMPs including EIC with / without stromal invasion measured less than 1 cm in the great diameter in all of the patients. The cytological features are summarized in Table 1 and representatively depicted in Figures 1, 2, and 3, including histological findings of three patients. The tumor diathesis was characterized as being predominantly mucous for 3 patients, watery for 3 patients, and bloody for 2 patients, but with no prominent necrotic inflammatory changes. The tumor cell clusters predominantly took a micropapillary pattern and occasionally a sheet-like pattern. Overlapping of the tumor cell nuclei was noted in a variable degree: often for 2 patients, and occasionally for 5 patients. There were varying amounts of normal epithelial cells, which served as a hallmark in distinction from the malignancy because of a significant difference in the nuclear size. Using Isis ver. 5 (MetaSystems GmbH, Altlussheim, Germany), the nuclear size (greatest diameter) of tumor cells were measured as well as normal atrophic cells in the cytological preparations. The numbers of counted cells for the former and the latter ranged from 50 to 70 in each case. On the total average of 8 cases, the tumor cell size was 12.5 μm and the relative ratio to the corresponding normal atrophic cells was 2.3 (Table 1). The chromatin was considerably dense, coarse or granular. The number of prominent nucleoli was as follows: 1 for 4 patients; 1 or 2 for 4 patients. Calcified deposits of psammoma body were not apparently observed in any patient.

**Table 1:** Cytological features of endometrial intraepithelial carcinomas

**Case**	**Age**	**Background**	**Cluster**	**Nucleus**		**Nucleolus**
		**Tumor diathesis**	**Structure**	**Overlapping**	^**a**^**Size: μm**	**Number**
1	61	watery	micropapillary	often	12.3 (2.3)	1~2
2	63	watery	micropapillary	often	11.3 (2.1)	1~2
3	76	bloody	sheet-like	occasional	12.8 (2.1)	1~2
4	60	watery	micropapillary	occasional	12.9 (2.3)	1
5	61	bloody	micropapillary	occasional	11.3 (2.4)	1
6	65	watery	sheet-like	rare	14.8 (2.8)	1
7	49	watery	micropapillary	occasional	10.6 (2.2)	1
8	62	watery	micropapillary	occasional	13.6 (2.2)	1~2

^a^: average of largest diameter of tumor cells (relative value compared to normal nucleus).

The clinicopathological profiles are summarized in Table 2. All the patients had an EMP in which atypical tumor cells were arranged predominantly in a tubular structure with micropapillary protrusions, replacing the pre-existing endometrial glands. Micropapillary protrusions and small tufts were accompanied with or without thin fibrovascular cores. These tumor cells showed conspicuous nuclear pleomorphism and hyperchromatism, frequent mitotic figures, high N/C ratio, and also one or two prominent nucleoli. The tumor front was abruptly made into normal glandular epithelium and surface-lining epithelium in the EMP. Minimal stromal invasion of the tumor cells limited to the EMP was recognized in 3 patients. In addition, similar tumor cells also were also found to replace some atrophic endometrial glands in 4 patients, lacking stromal invasion. In the background, cystic dilatation of atrophic normal glands was evident especially in the EMP.

**Table 2:** Clinicopathological profiles of endometrial intraepithelial carcinomas

**Case**	**EIC location**	**Immunohistochemistry**				**FIGO stage**	**Outcome (periode)**
		**P53**^**a**^	**MIB-1**^**b**^	**ER**^**c**^	**PgR**^**d**^		
1	polyp^h^	+	30	+	+	IA	DF^i^ (66 months)
2	polyp & endometrium	+	60	-	-	IA	DF (65 months)
3^f^	polyp & endometrium	+	35	-	-	IA	DF (36 months)
4	polyp	+	30	-	-	IA	DF (30 months)
5	polyp^h^ & endometrium	+	45	-	+	IA	DF (39 months)
6	polyp	+	25	-	-	IA	DF (39 months)
7 ^g^	polyp	-	30	-	-	IA	DOD^j^ (44 months)
8	polyp^h^ & endometrium	-	70	-	-	IA	DF (12 months)

^a^:+, strongly positive cells more than 80%.

^b^: index of positive cells(%), ^c^: +, positive cells more than 80%.

^d^: +, positive cells more than 80%.

^e^: since initial diagnosis.

^f^: history of breast cancer with Tamoxifen administration.

^g^: autopsy performed.

^h^: with stromal invasion, ^i^: disease free, ^j^: death on disease.

**Figure 4:**
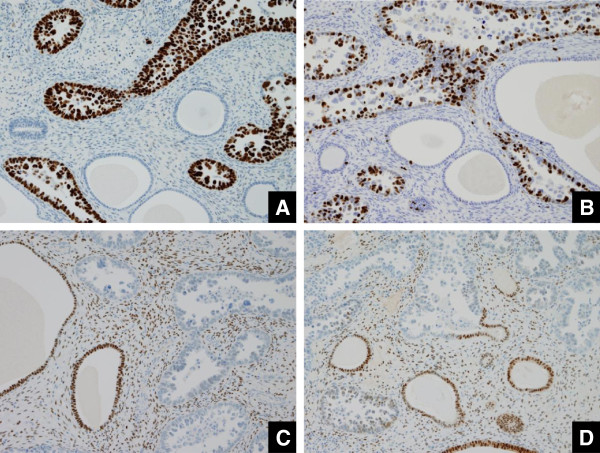
**Immunohistochemical staining profiles represented by Case 5 (A. p53 × 40, B. MIB-1 × 40, C. ER × 20, D. PgR × 20). A.** Almost all of the carcinoma cells are strongly positive. **B.** More than half the carcinoma cells are labeled. **C.** The expression is observed in the cystically dilated normal glands and stromal cells but not in the carcinoma cells. **D.** The expression behavior is basically similar to the ER expression.

Immunohistochemical expressions of p53 (DO7, 1:50, Dako, Glostrup, Denmark), Ki-67 (MIB-1, 1:100, Dako, Glostrup, Denmark), ER (SP-1, 1:1, Ventana, AZ, USA), and PgR (1E2, 1:1, Ventana, AZ, USA) were examined (Figure 4). Six patients showed marked p53 expression with a positive ratio of more than 80%. The labeling index of Ki-67 ranged from 25 to 70% (av. approximately 40). ER was mildly expressed in one patient and PgR expression also was mildly observed in 2 patients.

FIGO stage was determined at IA for all the patients. Neither adjuvant chemotherapy nor irradiation was performed. Seven patients have been taking an uneventful clinical course (follow-up period ranging from 12 to 66 months, av. 41), but one patient died of the recurrent disease massively occupying the abdominal cavity 44 months after the initial diagnosis.

## Discussion

Endometrial polyp is a common benign disease of the uterus. The overall incidence where cancer arises in the endometrial polyp has been reported as only a few percent, while postmenopausal women with EMP are at an increased risk of malignancy, compared to premenopausal women [10,13-15]. As risk factors for development of the malignancy in EMP, hypertension, obesity, and unopposed estrogen therapy have been indicated in addition to postmenopausal status [15]. There are unique or specific pathological and clinical features for EIC as a putative precursor of uterine body serous adenocarcinoma, as follows [1,4,5,7,16-20]: close association with an EMP; multicentric occurrence in the inactive or resting thin endometrium, lacking alteration in the architecture in the endometrium lacking an intervening phase of endometrial hyperplasia; extrauterine invasive extent, irrespective of an absence of stromal invasion of the uterus; and controversial outcome, whether taking a favorable or unfavorable clinical course. Development of EIC associated with an EMP may also be explained in part by Tamoxifen administration following breast cancer treatment [6,17]. However, little has been documented about the endometrial cytological features of minimal serous adenocarcinoma of the uterine body including EIC, mainly due to the fact that the disease is not frequently encountered in the routine practice. To the best of our knowledge, there is a case report cytologically referring to serous adenocarcinoma of the uterine body associated with an EMP [21], but here the patient already showed peritoneal dissemination at the initial presentation. In another report, serous adenocarcinomas of the uterine body were clinico-cytologically reviewed but they were all at advanced stages [22]. The latter report mentions that cytologically diagnostic findings of uterine body serous adenocarcinoma would be based on the following features: papillary cluster as well as tubular cluster, arborescent structure, frequent exfoliation, psammoma body, eccentric enlarged nuclei, coarsely granular chromatin, numerous nucleoli, and diameter of greater than G1 endometrioid adenocarcinoma [22]. In our review, 5 patients were purely non-invasive as they were composed of only EIC existing in an EMP, with few or no necroinflammatory changes. The background was found to be characteristically watery or mucous (Figure 1) in a way of simulating the immigration of extrauterine carcinoma cells via the fallopian tubes [12]. Many clusters were arranged in micropapillary architecture without fine vascular stroma, and others were occasionally tubular. An abrupt transition between the EIC cells and atrophic endometrial cells was noted by front formation (Figure 2). Conspicuous nucleoli were found in 3 patients (Figure 3). Even though it seems difficult to reach a definite diagnosis, these findings may serve as a diagnostic indicator for the uterine body serous adenocarcinoma, whether being at an early stage or an advanced stage.

Differential diagnoses for atypical epithelial sheets in the normal-appearing endometrial background include variable interpretations as follows: EIC, endometrial glandular dysplasia, endometrial intraepithelial neoplasia, hyperplastic polyp, and metastatic carcinoma [9,23]. In addition to these, isolated atypical glands with morphological and immunohistochemical features of a typical endometrial hyperplasia or type I endometrial adenocarcinoma may be encountered in grossly normal postmenopausal endometrium of asymptomatic patients [24]. Especially in the postmenopausal state, thin and smooth endometrium with atrophy is composed of hypocellular and flattened glands lined by cells with reduced or absent mitosis and sparse fibrous stroma [25]. It is supposed that the abrupt transition of the atrophic endometrial cells to atypical cells may indicate EIC in postmenopausal women.

Overexpression of p53 is closely linked to the rapid growth of uterine body serous adenocarcinoma [6]. In EIC, too, frequent p53 mutation is reported to be associated with LOH of 17p [26]. Thus, immunohistochemistry for p53 is also adjunctively available in the diagnosis of uterine body serous adenocarcinoma, irrespective of whether it is at an early stage or advanced stage [27], in the discrimination between uterine body serous adenocarcinoma and benign morphologic mimics [28]. Negativity of ER and PgR staining is more reasonable with the diagnosis of serous adenocarcinoma including EIC.

In summary, EMP arising in postmenopausal women is at a high risk of giving birth to the tumorigenetic background for endometrial carcinomas such as endometrioid adenocarcinoma and serous adenocarcinoma including EIC. Endometrial cytology may become a helpful diagnostic approach for serous adenocarcinoma, whether it is at early or advanced stage, from the viewpoint of the distinctive cytological features. Above all, it should be noted that some EIC behaves in a more aggressive fashion regardless of whether or not it lacks apparent stroma invasion and extrauterine extent.

## Patients’ consents

Written informed consents were obtained from the reviewed patients for publication of this report and any accompanying images.

## Ethical approval

This review was performed based on compliance with the Helsinki Declaration.

## Competing interests

All of the authors declare that they have no competing interests.

## Authors’ contributions

MY conducted the method of reviewing the cases. TK took part in analysis of cytological findings. SH analyzed the clinical information. KS took part in measurement of the nuclear size. KS took part in measurement of the nuclear size. MM analyzed the clinical information. MN performed immunohistochemistry. SM assisted in immunohistochemistry. ON designed tables and figures. YK searched the literature. All authors read and approved the final manuscript.
